# Comprehensive investigation of tumor immune microenvironment and prognostic biomarkers in osteosarcoma through integrated bulk and single-cell transcriptomic analysis

**DOI:** 10.3389/fimmu.2024.1424950

**Published:** 2024-07-23

**Authors:** Shaoyan Shi, Li Zhang, Xiaohua Guo

**Affiliations:** Honghui Hospital, Xi’an Jiaotong University, Xi’an Honghui Hospital North District, Xi’an, Shanxi, China

**Keywords:** osteosarcoma, tumor microenvironment, transcriptome sequencing, prognostic model, single-cell sequencing

## Abstract

Osteosarcoma (OS) is an aggressive and highly lethal bone tumor, highlighting the urgent need for further exploration of its underlying mechanisms. In this study, we conducted analyses utilizing bulk transcriptome sequencing data of OS and healthy control samples, as well as single cell sequencing data, obtained from public databases. Initially, we evaluated the differential expression of four tumor microenvironment (TME)-related gene sets between tumor and control groups. Subsequently, unsupervised clustering analysis of tumor tissues identified two significantly distinct clusters. We calculated the differential scores of the four TME-related gene sets for Clusters 1 (C1) and 2 (C2), using Gene Set Variation Analysis (GSVA, followed by single-variable Cox analysis. For the two clusters, we performed survival analysis, examined disparities in clinical-pathological distribution, analyzed immune cell infiltration and immune evasion prediction, assessed differences in immune infiltration abundance, and evaluated drug sensitivity. Differentially expressed genes (DEGs) between the two clusters were subjected to Gene Ontology (GO) and Gene Set Enrichment Analysis (GSEA). We conducted Weighted Gene Co-expression Network Analysis (WGCNA) on the TARGET-OS dataset to identify key genes, followed by GO enrichment analysis. Using LASSO and multiple regression analysis we conducted a prognostic model comprising eleven genes (ALOX5AP, CD37, BIN2, C3AR1, HCLS1, ACSL5, CD209, FCGR2A, CORO1A, CD74, CD163) demonstrating favorable diagnostic efficacy and prognostic potential in both training and validation cohorts. Using the model, we conducted further immune, drug sensitivity and enrichment analysis. We performed dimensionality reduction and annotation of cell subpopulations in single cell sequencing analysis, with expression profiles of relevant genes in each subpopulation analyzed. We further substantiated the role of ACSL5 in OS through a variety of wet lab experiments. Our study provides new insights and theoretical foundations for the prognosis, treatment, and drug development for OS patients.

## 1 Introduction

OS is the predominant primary malignant bone tumor, representing 20%-40% of all bone cancers (1). Globally, the annual incidence is approximately 1–3 cases per million individuals (2), with around 800 new cases diagnosed annually in the United States (3). Among children and adolescents, OS has the highest incidence, with a median age of 18 years, positioning it as the third most prevalent malignant tumor within this demographic (4, 5). The disease demonstrates a bimodal distribution, with the initial peak typically manifesting during adolescence (with an average age of 10–14 years for females and 15–19 years for males), followed by a second peak occurring after the age of 65 (6). Among OS patients, the 5-year relative survival rates are approximately 60% for individuals under 30 years old, 50% for those aged 30–49, and diminish to 30% for patients aged 50 or older (7). OS commonly affects the long bones of the limbs, whereas tumors in the chest and pelvic bones present a higher risk of metastasis OS (8). Chemical agents such as methylcholanthrene, beryllium oxide, and zinc beryllium silicate are potential inducers of OS, along with radiation exposure, electrical burns, and genetic factors (9). Gender and race significantly influence the incidence of OS. Males are more frequently affected than females across all age groups. Additionally, the highest incidence rates are observed among black individuals (10). Survival rates are highly correlated with tumor location and staging (11). Tumors detected at an earlier stage and located in more accessible regions generally have a better prognosis compared to those found at advanced stages or in less accessible areas such as the pelvis or chest. These factors underscore the importance of early detection and tailored treatment strategies for improving patient outcomes.

For suspected OS patients, initial cost-effective screening involves X-ray examinations, followed by CT or MRI scans to further evaluate tumor involvement (12). The standard treatments for OS encompass neoadjuvant multidrug chemotherapy, typically involving cisplatin, doxorubicin, methotrexate (commonly known as MAP therapy), and ifosfamide. This is typically followed by surgical intervention and subsequent postoperative chemotherapy (13). Despite its rarity, OS carries a poor prognosis, with surgical intervention being the primary curative treatment; however, patients undergoing surgery alone have a survival rate of only about 15% (14). For patients who are not candidates for surgical resection or those with residual tumors at the resection margins, as well as for OS patients with poor response to chemotherapy, radiation therapy serves as an effective method for local control and symptom relief (15). Additionally, many OS patients have small lung metastases at diagnosis. The 5-year survival rate is over 78% for localized disease but falls to 25% for metastatic or recurrent OS (16). Metastatic OS is highly invasive with a poor prognosis, emphasizing the urgent need for early diagnosis and targeted therapy. Continued investigation into the mechanisms underlying the onset and progression of OS, especially those contributing to elevated recurrence and metastasis rates, is paramount. The identification of pivotal biomarkers and exploration of essential target genes are crucial for enhancing the diagnostic, therapeutic, and prognostic approaches for OS.

Currently, numerous studies have investigated the role and mechanisms of specific gene families in OS (17, 18), such as the presence of pro-inflammatory FABP4^+^ macrophage infiltration observed in pulmonary metastatic OS lesions. In comparison with primary osteoblastic OS lesions, sub-osteoclast infiltration has been observed in chondroblastic, recurrent, and pulmonary metastatic OS lesions OS (19). It has been suggested that TME promotes tumor cell proliferation and immune evasion (20), yet its value as an immunotherapeutic target in OS remains unknown (21). We analyzed bulk transcriptome sequencing data of OS and healthy control samples, as well as single cell sequencing data, obtained from public databases. Initially, we assessed the expression differences of four TME-related gene sets between tumor and control groups. Next, we performed unsupervised clustering analysis on tumor tissues, identifying two distinct clusters. We calculated the GSVA score differences of the four TME-related gene sets between Clusters 1 (C1) and 2 (C2) and conducted single-variable Cox analysis. For the two clusters, we analyzed survival rates, clinical-pathological distribution differences, immune cell infiltration, immune evasion, immune cell abundance, and drug sensitivity. DEGs of the two clusters were subjected to GO and GSEA. We performed WGCNA on the TARGET-OS dataset to identify key genes, followed by GO enrichment analysis. Using LASSO and multiple-factor regression analysis, we constructed a prognostic model comprising eleven genes (ALOX5AP, CD37, BIN2, C3AR1, HCLS1, ACSL5, CD209, FCGR2A, CORO1A, CD74, CD163). The model demonstrated good diagnostic performance and prognostic evaluation potential in both training and validation cohorts. Next, using the model, we conducted immune analysis, drug sensitivity analysis, and enrichment analysis. In single-cell sequencing, we performed dimensionality reduction and annotated cell subtypes, followed by analyzing the expression of relevant genes in each subtype. We further validated the potential impact of ACSL5 inhibition on the invasive behavior of OS cells through various wet lab experiments. Our study offers novel insights and a theoretical framework for the prognosis, treatment, and drug development targeting OS patients.

## 2 Material and methods

### 2.1 Data acquisition and preprocessing

In this study, we used the “TCGAbiolinks” R package to retrieve bulk transcriptomic data of OS from the public database The Cancer Genome Atlas (TCGA, https://portal.gdc.cancer.gov/), specifically the TARGET-OS dataset comprising 86 patients. We obtained healthy control bulk transcriptomic data from the public database Genotype-Tissue Expression (GTEx, www.org/home/index.html). Additionally, we downloaded bulk transcriptomic datasets of OS, including GSE21257 (53 patients) and GSE16091 (34 patients), as well as single cell sequencing datasets GSE162454 and GSE198896, from the public database GEO (https://www.ncbi.nlm.nih.gov/geo/). We excluded samples with missing information from the analysis. Using the “ComBat” function from the “sva” package, we standardized the TARGET-OS and GTEx datasets into bulk matrices in Transcripts per million (TPM) formats. All open-access public databases utilized in this study allow unrestricted access and utilization without the need for additional ethical approval. Our data retrieval and analysis processes adhered to relevant regulations.

### 2.2 Investigation of expression levels of tumor microenvironment-related gene sets

Utilizing the “signature_collection” function of the “IOBR” package, we identified four TME-related gene sets (TMEscoreA_CIR, TMEscoreB_CIR, TMEscoreA_plus, and TMEscoreB_plus) within the merged dataset of TARGET-OS/GTEx. Subsequently, we employed heatmaps to illustrate the expression disparities of these four relevant gene sets between the tumor and normal groups.

### 2.3 Hierarchical clustering and TME landscape analysis

We used the “ConsensusClusterPlus” package to perform unsupervised clustering analysis on tumor tissues. The optimal number of clusters (k) was determined by scoring and evaluating matrix plots, Cumulative Distribution Function (CDF) curves, and Proportion of Ambiguous Clustering (PAC) curves. High intra-cluster cohesion, low inter-cluster coupling, a smooth CDF curve, and the lowest PAC curve value are included as our selection criteria. Following multiple standard screenings, we identified two significantly different clusters. Using the “GSVA” package, we calculated scores for four TME-related gene sets in the TARGET-OS dataset (GSVA: gene set variation analysis for microarray and RNA-seq data). We used heatmaps and box plots to show the differences in GSVA scores of the four TME-related gene sets between Clusters 1 (C1) and 2 (C2). We then performed univariate Cox analysis on these gene sets and displayed the differences in hazard ratios with forest plots. Additionally, we conducted survival analysis on the two clusters and displayed the prognostic differences using Kaplan-Meier curves. We used a stacked bar graph to show the compositional differences in clinical pathological information such as Age and Stage between the two clusters.

We utilized five immune cell infiltration prediction algorithms (CIBERSORT, TIMER, MCPcounter, EPIC, quanTIseq) to assess the immune cell infiltration status of the two clusters and visualized the results using box plots. From the TISIDB database (http://cis.hku.hk/TISIDB/), we downloaded 150 immunomodulators and chemokines, including 41 chemokines, 24 immunoinhibitors, 46 immunostimulators, 21 MHC molecules, and 18 receptors. Furthermore we generated a heatmap to illustrate the expression differences of immune modulators between the two clusters. We employed the TIDE (Tumor Immune Dysfunction and Exclusion) database (http://tide.dfci.harvard.edu/) to predict tumor immune escape via immune checkpoint analysis for the two clusters. We visualized the different responses of the two clusters to immune checkpoints using stacked bar graphs. Additionally, we assessed the efficacy of immune checkpoint blockade (ICB) through TIDE scoring. Three immune-suppressive cell types (MDSCs, TAM.M2, and CAFs) were selected, and violin plots were employed to demonstrate the differences in immune-suppressive cell infiltration abundance between the two clusters. Additionally, using the “OncoPredict” package, we predicted the sensitivity of the two clusters to four drugs (Bortezomib, XAV939, Selumetinib, Trametinib).

### 2.4 Enrichment analysis and weighted gene co-expression network analysis

We employed the “limma” package to identify DEGs between the two clusters and visualized the upregulated and downregulated genes using volcano plots. Subsequently, we conducted GO enrichment analysis on the DEGs and presented bar graphs showing the top ten pathways in each of the BP, CC, and MF subclasses. Following this, we performed GSEA on the DEGs and demonstrated the downregulated pathways within the C2 category. For the TARGET-OS dataset, we conducted WGCNA, selecting appropriate soft thresholds based on Scale Independence and Mean Connectivity. Utilizing the optimal soft threshold, we constructed a co-expression network, partitioned genes into modules, and depicted a Cluster Dendrogram for visualization. We computed the correlation between modules and clinical traits (futime, fustat, age, stage, cluster), illustrating the correlation heatmap. We identified the module most correlated with the cluster as the key module and selected key genes within this module based on criteria of MM (module membership) > 0.6 and GS (gene significance) > 0.3. We then performed GO enrichment analysis on these key genes.

### 2.5 Construction and validation of machine learning prognostic models

We chose the TARGET-OS dataset as the training set, while two GEO datasets served as validation sets. Utilizing the Least Absolute Shrinkage and Selection Operator (LASSO) and multiple regression analysis, we constructed the prognostic model (22). In the TARGET-OS dataset, we identified prognostic hub genes by obtaining the optimal parameter λ. Through multiple Cox regression analysis, we determined the coefficients of each gene in the model and visualized them using bar graphs. We determined the risk score of the model by summing the product of the expression level of each gene and its respective coefficient.


$$\text{Risk score}=\sum_{i=1}^{n}\left[Exp_{gene_{i}}*\beta_{i}\right]$$

In this context, $Exp_{gene_{i}}$ denotes the expression level of the model gene, and $\beta_{i}$ represents the coefficient corresponding to the model gene. We divided the training and validation sets into high-risk and low-risk groups based on the median score of each dataset. We then observed survival differences over time between these groups in all three datasets. Additionally, we conducted ROC curve analysis to evaluate the model’s performance at 1-year, 3-year, and 5-year intervals.

### 2.6 Model-based immune analysis, drug sensitivity analysis, and enrichment analysis

Immune cell infiltration analysis was conducted using the CIBERSORT algorithm to identify relevant immune cell subtypes. Box plot analysis was conducted to evaluate the distribution disparities among various subtypes across two risk groups. Additionally, we utilized box plots to illustrate the expression differences of M2 markers and depleted T cell markers between the two risk groups. We obtained the data from the Progenitor Cell Biology Consortium (PCBC, https://www.synapse.org), and violin plots were generated to display the mRNAsi index of two risk groups, allowing for an analysis of cellular stemness differences between the two risk groups. We analyzed the sensitivities of nine drugs in the two risk groups. Following this, we conducted differential gene expression analysis between the groups and used GSEA to identify dysregulated pathways.

### 2.7 Single-cell sequencing analysis

Analysis of single cell sequencing data was conducted utilizing both the “Seurat” package and the “SCP pipeline.” Data underwent quality control and data cleaning to ensure the accuracy and reliability of subsequent analyses, with quality control criteria set as follows: nFeature_RNA< 9000, percent.mt< 25. We used the harmony method for batch correction of data across multiple samples. Utilizing the Uniform Manifold Approximation and Projection (UMAP) method, we performed dimensionality reduction on the integrated single-cell sequencing data. We annotated ten major cell subtypes using specific markers for each and visualized them. To show the correlation between these subtypes and individual genes, we created violin plots. We used SingleR for automatic annotation and CopyKAT to identify malignant cells. We computed the upregulated and downregulated genes in each cell subtype and displayed the top five of each in volcano plots. GO_BP analysis was conducted, with dot plots illustrating the enriched upregulated pathways in each cell subtype. Using Seurat, we evaluated the activity level of prognostic models in the single-cell dataset.

### 2.8 Cell culture and siRNA transfection

We acquired OS cell lines MG63 and Saos-2 from Procell Life Science & Technology, China, and cultured in Dulbecco’s Modified Eagle’s Medium (DMEM) containing 10% fetal bovine serum (FBS) at 37°C in a humidified atmosphere with 5% carbon dioxide. Small interference RNA (siRNA) targeting ACSL5 was transfected using sequences sourced from HANBIO, China, and LipoFiter 3.0 (HANBIO, China) was employed for the transfection process. The specific sequences for siRNA were as follows:

si-NC Sense: UUCUCCGAACGUGUCACGUTT;

Antisense: ACGUGACACGUUCGGAGAATT;

Si-ACSL5–1 Sense: CAAATACTTTCGGACCCAAA;

Antisense: CTCTTCTTGACCTGAACAAT;

Si-ACSL5–2 Sense: CATGATAGTTTCTGGGACAA;

Antisense: CCAAGTTGTAAGGGAAGCCAT

### 2.9 Real-time PCR

Total RNA extraction utilized Trizol reagent (Solarbio Science & Technology, China), with subsequent reverse transcription into cDNA employing a two-step RNA reverse transcription kit (TaKaRa Bio Inc., Japan). The RT-qPCR reaction comprised cDNA, RT-qPCR SYBR Green (TaKaRa Bio Inc., Japan), and primers, following cyclic parameters: initial denaturation at 95°C for 30 seconds, succeeded by 40 cycles of denaturation at 95°C and annealing at 60°C for 34 seconds. The primers used for cDNA amplification were as follows: ACSL5-F: 5′-GGCATTGGTGCTGATAGG-3′ and ACSL5-R: 5′-TCTTCTCCCCTCTTTGCTT-3′; β-actin-F: 5′-CAAGAGATGGCCACGGCTGCT-3′ and β-actin-R: 5′-TCCTTCTGCATCCTGTCGGCA-3′ (23, 24).

### 2.10 Cell proliferation assay

We assessed cell viability employing the CCK-8 kit (Seven, China). we seeded a 96-well plate with a single-cell suspension at a density of 5 × 10^3^ cells per well. Subsequently, we added 10 μL of CCK-8 solution to each well every 24 hours, and the plate was then incubated for 2 hours. (25) We measured the optical density (OD) at 450 nm using a multifunctional enzyme-linked immunosorbent assay reader (Synergy H1, BioTek, USA).

### 2.11 Cell migration and invasion assays

We evaluated cell migration and invasion utilizing transwell chambers featuring an 8.0-μm pore size (Corning, USA). We loaded the lower chamber with 500 μL of medium supplemented with 10% FBS, while 2 × 10^4^ cells in serum-free medium were seeded onto the upper chamber. For invasion assessments, we coated the transwell membrane with 1 mg/ml Matrigel. After a 24-hour incubation at 37°C, we gently removed the non-migrated cells using cotton swabs. Cells that migrated to or invaded the underside of the membrane were stained with crystal violet and quantified. Three randomly chosen microscopic fields were tallied for each well.

### 2.12 EdU assay

We assessed the proliferation capacity of cells post-knockdown using the EdU Cell Proliferation Assay Kit (Beyotime, China). We subjected MG63 and Saos-2 cells to knockdown and cultured them on six-well plate. We prepared a 2x EdU working solution in serum-free medium using a 10 mM EdU solution, preheated it, and mixed it with the culture medium to obtain a 1x EdU solution. The cells were incubated with this solution for 12 hours. After incubation, we fixed the cells with 2.5 mL of PBS containing polyformaldehyde for 15 minutes at room temperature. We then washed the cells three times with 2.5 mL of PBS for 5 minutes each. Next, we treated the cells with 2.5 mL of permeabilization buffer for 20 minutes at room temperature. After removing the permeabilization buffer, we washed the cells twice with 2.5 mL of PBS and then removed the washing solution. We prepared Click-iT reaction mixture and added to each slide (100 µl), followed by incubation in the dark at room temperature for 30 minutes. Subsequently, we removed the reaction mixture, and each well was washed once with 2.5 mL of PBS, followed by removal of the washing solution. We also performed DAPI nuclear staining and captured images under a microscope for further analysis.

### 2.13 Colony-formation assays

Cells in logarithmic growth phase were trypsinized, resuspended in complete culture medium supplemented with 10% fetal bovine serum, and counted. We seeded MG63 and Saos-2 cells in six-well plates at a density of 700 cells per well and cultured for 14 days, with media changes every 3 days and continuous monitoring of cell status. We captured images of the cells under a microscope, followed by a single wash with PBS. Cells were then fixed with 1 mL of 4% polyformaldehyde per well for 30 minutes, washed once with PBS, and stained with crystal violet solution (Beyotime, China) for 10 minutes. After several washes with PBS, the cells were air-dried and photographed.

### 2.14 Statistical analysis

We performed statistical analyses using R software (version 4.1.3). For single cell sequencing analysis, we used the “Seurat” package and “SCP pipeline.” We used the “IOBR” package for gene set acquisition and immune infiltration analysis, and “oncoPredict” for drug sensitivity analysis. We employed “SingleR” for cell annotation and “copykat R” for tumor cell identification. We used the “ConsensusClusterPlus” package for unsupervised clustering analysis, and “clusterProfiler” for enrichment analysis. Differential expression analysis was implemented using the “limma” package. A threshold of p< 0.05 was considered statistically significant (* p< 0.05; ** p< 0.01; *** p< 0.001; **** p< 0.0001).

## 3 Results

### 3.1 Exploration of expression levels of tumor microenvironment-related gene sets

**Figure 1:**
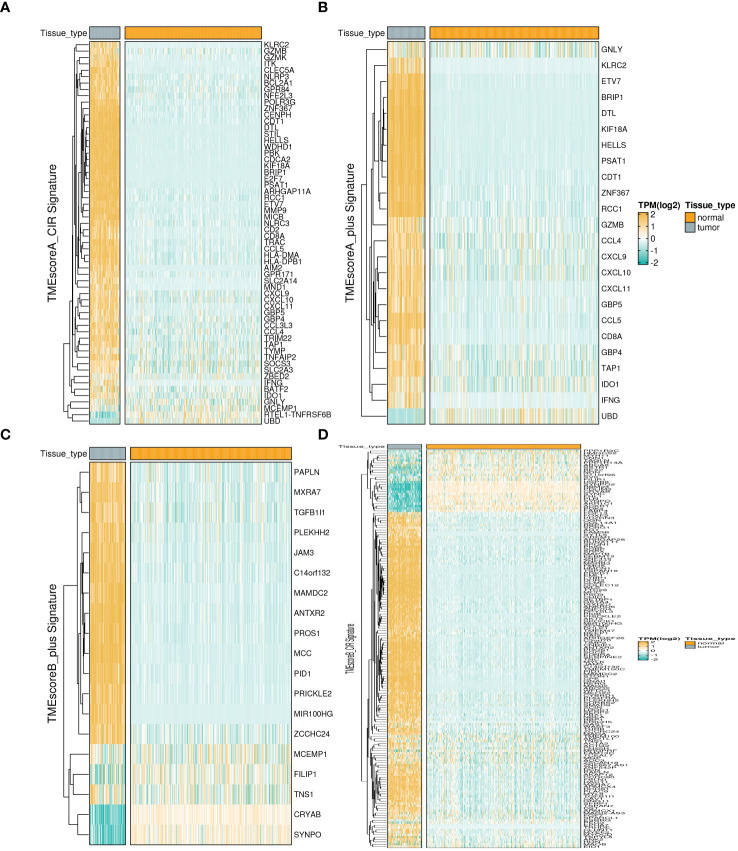
Expression profiles of TME-related Signatures in OS. The expression patterns of four TME-related signatures including TMEscoreA_CIR **(A)**, TMEscoreA_plus **(B)**, TMEscoreB_CIR **(C)**, and TMEscoreB_plus **(D)**.

We analyzed the expression differences of four TME-related gene sets (TMEscoreA_CIR, TMEscoreB_CIR, TMEscoreA_plus, and TMEscoreB_plus) between tumor and normal groups using integrated bulk expression matrices from the TARGET-OS and GTEx datasets. Heatmaps illustrate that most genes within the four gene sets exhibit significantly higher expression in the tumor group (**Figures 1A–D**).

### 3.2 Hierarchical clustering and TME landscape analysis

**Figure 2:**
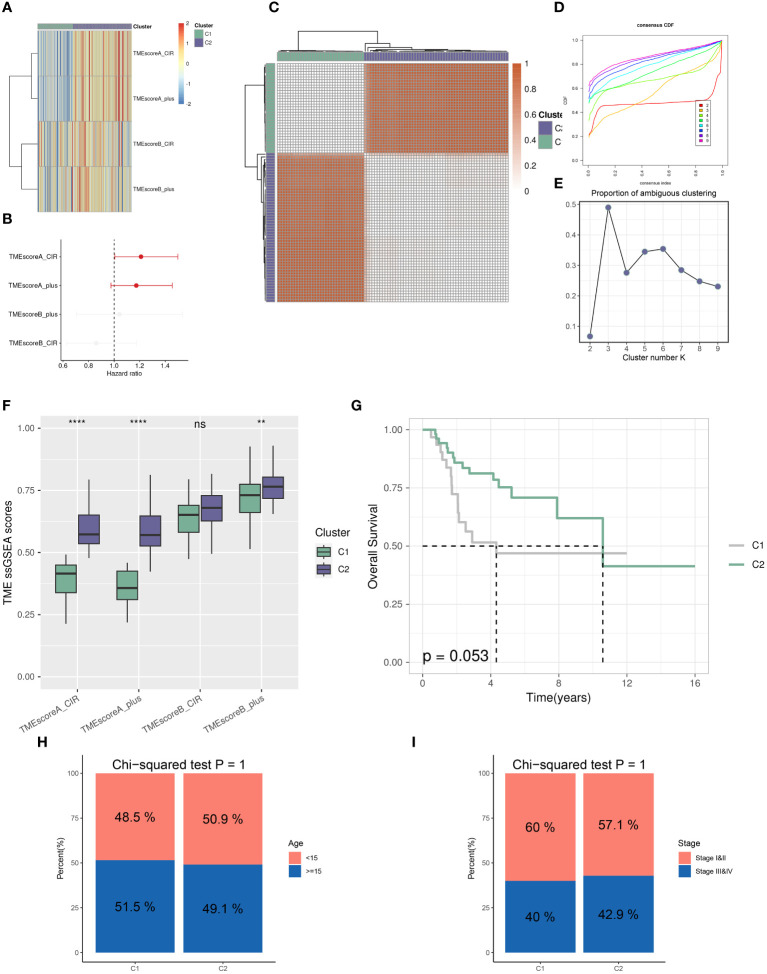
Distinct TME landscapes in OS. **(A)** The GSVA scores of each TME-related signature between two TME subclusters. **(B)** Forest plot illustrating the hazard ratio of each TME-related signature determined by Univariate Cox regression analysis. **(C)** The consensus score matrix of glioma samples in TARGET-OS when the clustering number k = 2. The consensus score represents the intensity of interaction between two samples. **(D, E)** The CDF curves **(D)** and PAC scores **(E)** of the consensus matrix for each (k) **(F)** Boxplots showing the distribution of GSVA scores of each TME-related signature between two TME subclusters. **(G)** The survival differences between two TME subclusters, analyzed by Kaplan-Meier curves with the log-rank test. **(H, I)** Stacked Bar plots illustrating the distributions of age populations **(H)** and stages **(I)** between two TME subclusters. P values were calculated by the Chi-squared tests. ** p<0.01; **** p<0.0001.

**Figure 3:**
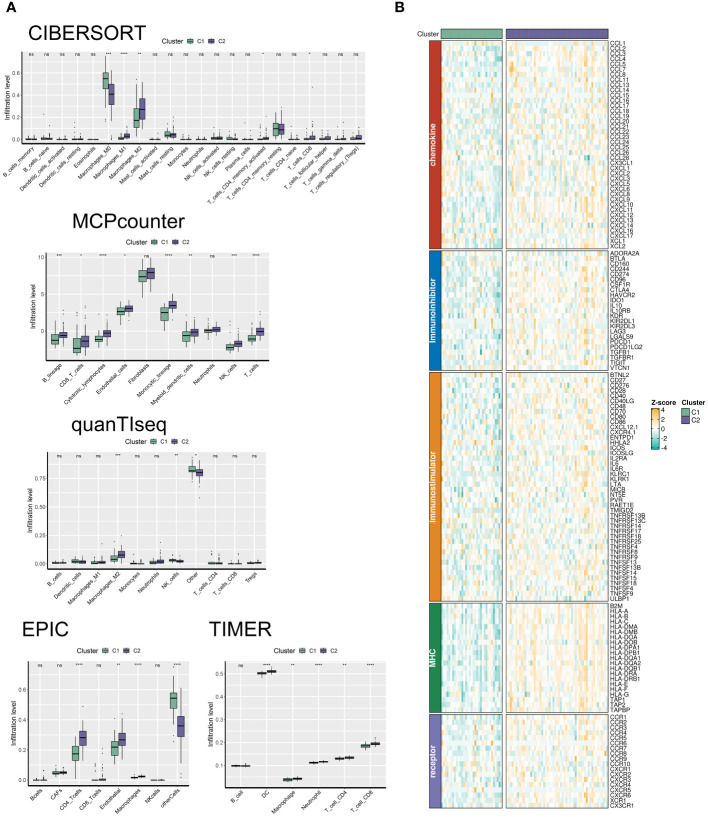
The C2 TME subcluster shapes a hot-TME in OS. **(A)** The infiltration abundances of immune cell subsets evaluated by CIBERSORT, MCP-counter, quanTIseq, EPIC, and TIMER for two TME subclusters. **(B)** The expression patterns of immunoregulators for two TME subclusters. * p<0.05; ** p<0.01; *** p<0.001; **** p<0.0001.

**Figure 4:**
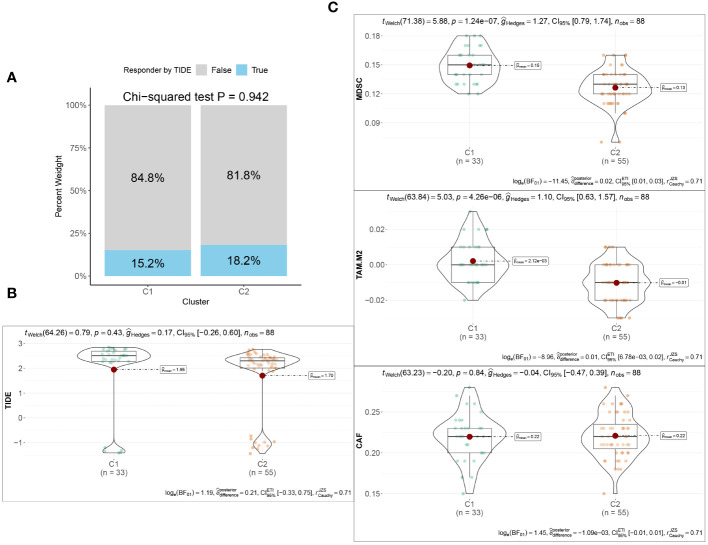
Immunotherapeutic response between the two TME subclusters. **(A)** Stacked Bar plots illustrating the distributions of predicted ICB responders between the two TME subclusters. **(B)** The TIDE scores between the two TME subclusters. **(C)** Violin plots showing the infiltration abundances of MDSC, M2-TAM, and CAF between the two TME subclusters.

**Figure 5:**
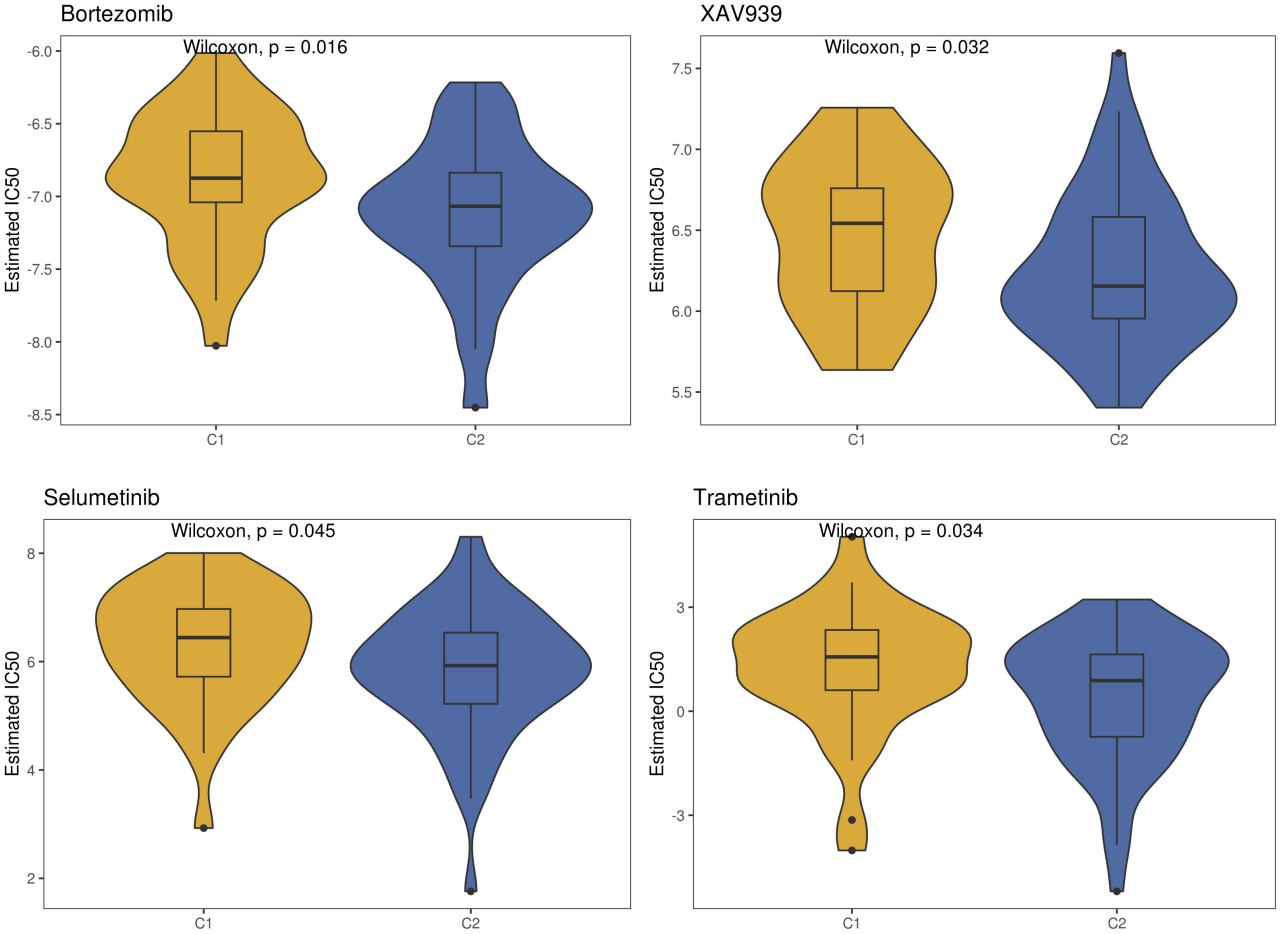
Drug sensitivity between the two TME subclusters.

Utilizing the “ConsensusClusterPlus” package, we conducted unsupervised clustering analysis on tumor tissues based on scoring. We computed GSVA scores for four TME-related gene sets in the TARGET-OS dataset and presented heatmap illustrations depicting score disparities of these genes between clusters C1 and C2. Notably, TMEscoreA_CIR and TMEscoreA_plus exhibited significantly higher scores in C2 compared to C1, whereas differences in scores for TMEscoreB_CIR and TMEscoreB_plus between the two groups were minimal (**Figure 2A**).Univariate Cox analysis was performed on the four TME-related gene sets, revealing significantly higher hazard ratios for TMEscoreA_CIR and TMEscoreA_plus compared to TMEscoreB_CIR and TMEscoreB_plus, indicating an unfavorable prognosis associated with the scores of TMEscoreA_CIR and TMEscoreA_plus (HR>1, **Figure 2B**). The matrix plot indicates high intra-cluster cohesion and low inter-cluster coupling (**Figure 2C**). Results for cluster numbers ranging from k=2 to k=9 were demonstrated, with k=2 showing a smooth CDF curve (**Figure 2D**) and the lowest PAC score (**Figure 2E**), thus suggesting k=2 as the optimal cluster number. Boxplots based on GSVA scores demonstrated that, except for TMEscoreB_CIR, scores for each TME-related gene set were higher in cluster C2 than in C1, with TMEscoreA_CIR and TMEscoreA_plus showing particularly significant differences (**Figure 2F**). Survival analysis was conducted on clusters C1 and C2, with KM curves illustrating a superior prognosis for C2 compared to C1 (**Figure 2G**). Additionally, stacked bar plots depicted distribution disparities of C1 and C2 across different clinical-pathological parameters. While patients aged over 15 years were slightly more predominant in C1 compared to C2 in terms of age distribution (**Figure 2H**), C2 exhibited a higher proportion of late-stage cases than C1 based on tumor stage distribution (**Figure 2I**). For immune cell infiltration analysis, we employed five algorithms, namely CIBERSORT, TIMER, MCPcounter, EPIC, and quanTIseq. Boxplots revealed that in CIBERSORT analysis, most immune cell infiltration levels were lower in cluster C1 compared to C2, whereas the infiltration level of Macrophages_M0 was lower in C2 than in C1. Results from MCPcounter indicated that the infiltration levels of various cell types were significantly higher in C2 than in C1. In quanTIseq analysis, the difference in infiltration levels between the two groups was minimal overall, but in the “Other” category, infiltration levels were slightly higher in C1 than in C2. EPIC analysis showed that CD4_Tcells and Endothelial cell infiltration levels were higher in C2 than in C1, while in the “OtherCells” category, infiltration levels were higher in C1 than in C2. TIMER analysis showed similar infiltration levels between the two groups, with C2 being higher than C1 in most cases (**Figure 3A**). Heatmaps of immunomodulators and chemokines along with the two clusters demonstrated higher expression of these 150 immunomodulators and chemokines in C2 (**Figure 3B**). We predicted tumor immune escape by examining immune checkpoints in clusters C1 and C2, with a stacked bar graph showing a higher response to immune checkpoints in C2 compared to C1 (**Figure 4A**). Violin plots displaying TIDE scores for the two clusters showed no statistically significant differences (**Figure 4B**).We selected three immune-suppressive cell types, CAFs, MDSCs, and TAM.M2, and presented violin plots illustrating the abundance of immune-suppressive cells between the two groups. In MDSCs and TAM.M2, infiltration was higher in C1 than in C2, with TAM.M2 showing particularly significant differences, while there was no significant difference between C1 and C2 in CAFs (**Figure 4C**).Furthermore, we conducted drug sensitivity analysis on four relevant drugs, Bortezomib, XAV939, Selumetinib, and Trametinib, for clusters C1 and C2. The IC50 values for all four drugs were lower in C2 than in C1, indicating higher sensitivity and better drug efficacy in C2 (**Figure 5**).

### 3.3 Enrichment analysis and weighted gene co-expression network analysis

**Figure 6:**
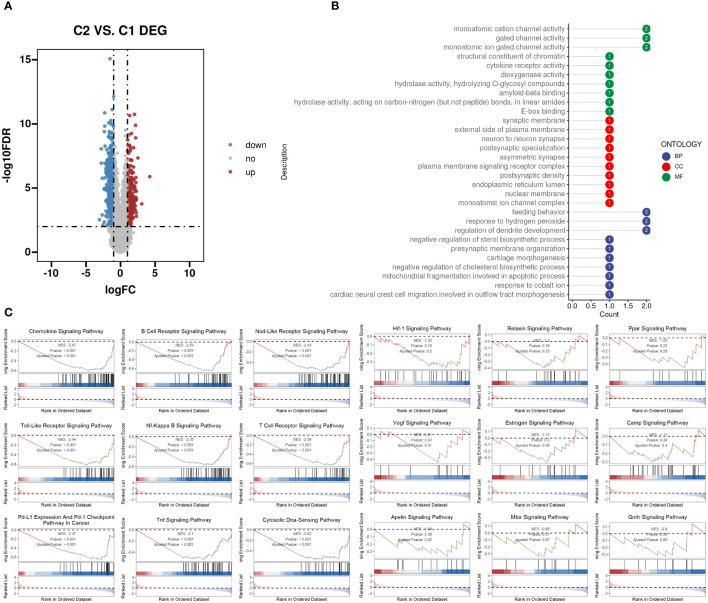
DEGs between the two TME subclusters. **(A)** Volcano plot showing the upregulated (colored in red) and downregulated (colored in blue) genes between the two TME subclusters. **(B)** Top ten enriched GO terms of hub genes. **(C)** GSEA of dysregulated pathways in the C2 TME subcluster.

**Figure 7:**
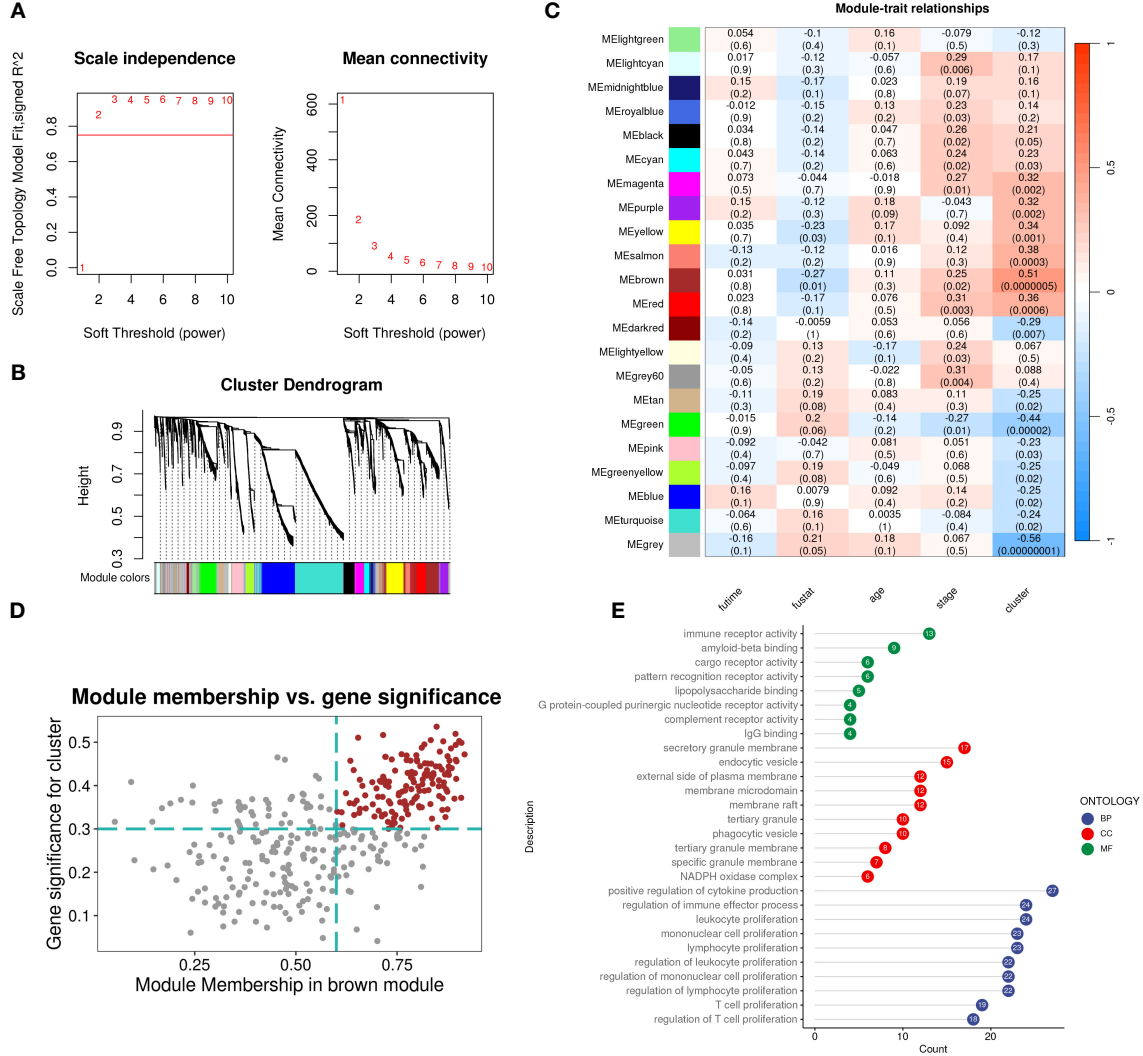
WGCNA identifies subcluster-related modules and hub genes inside. **(A)** Analysis of network topology for different soft-threshold power. The left panel shows the impact of soft-threshold power (power = 3) on the scale-free topology fit index; the right panel displays the impact of soft-threshold power on the mean connectivity. **(B)** Cluster dendrogram of the coexpression modules. Each color indicates a co-expression module. **(C)** Module-trait heatmap displaying the correlation between module eigengenes and clinical traits. **(D)** Correlation between module membership and gene significance in the brown modules. Dots in color were regarded as the hub genes of the corresponding module (MM > 0.6 & GS > 0.3). **(E)** Top ten enriched GO terms of hub genes.

We evaluated the differences between groups C1 and C2 in two directions: differential fold change and differential significance level. We identified DEGs between the two groups and displayed upregulated and downregulated genes using volcano plots (**Figure 6A**). Next, we performed GO enrichment analysis on the DEGs and illustrated the top ten pathways in the BP, CC, and MF categories using lollipop plots. In the MF category, the DEGs were enriched in pathways like gated channel activity, monoatomic cation channel activity, and monoatomic ion gated channel activity. The CC category showed consistent enrichment levels. In the BP category, the genes were enriched in pathways related to feeding behavior, response to hydrogen peroxide, and regulation of dendrite development. (**Figure 6B**). Furthermore, we performed GSEA on the DEGs, displaying the downregulated pathways in C2 (**Figure 6C**). Subsequently, we applied WGCNA to the TARGET-OS dataset, determining an appropriate soft threshold based on Scale Independence and Mean Connectivity (**Figure 7A**). Using the optimal soft threshold, we constructed a co-expression network, partitioned genes into modules, and visualized a dendrogram for clustering (**Figure 7B**). We computed the correlation between modules and clinical traits, depicting the results in a heatmap. The correlation between modules and the futime trait was generally low with minimal variation, while more modules exhibited negative correlations with the fustat trait. Modules were mainly positively correlated with age and stage. The MEbrown module had a strong positive correlation with the cluster trait, while the MEgrey module had a strong negative correlation. (**Figure 7C**). We identified the MEbrown module, which had the highest correlation with the cluster trait, as the key module. Subsequently, we filtered out key genes of the module based on Module Membership (MM) and Gene Significance (GS) criteria (MM > 0.6 & GS > 0.3) (**Figure 7D**). We conducted GO enrichment analysis on the key genes, revealing enrichment in pathways such as immune receptor activity in MF, secretory granule membrane in CC, and positive regulation of cytokine production in BP. Overall, the number of genes enriched in BP pathways was significantly higher than those in MF and CC pathways (**Figure 7E**).

### 3.4 Construction and validation of machine learning prognostic model

**Figure 8:**
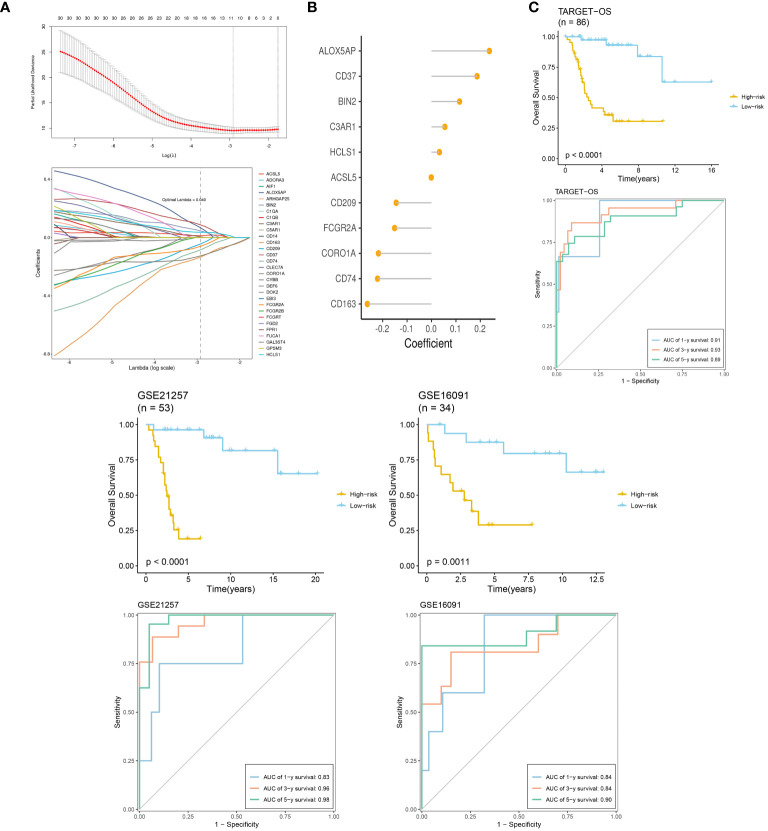
TME-related prognostic signature construction and validation. **(A)** The selection of prognostic hub genes based on the optimal parameter λ that was obtained in the LASSO regression analysis. **(B)** Lollipop chart of the coefficients of signature genes determined by the multiCox regression analysis. **(C)** Survival differences between two groups in the three datasets. Time-dependent ROC analysis of the model in the three datasets.

We selected TARGET-OS as the training set and two GEO datasets as the validation sets. LASSO and multivariable Cox regression analyses were performed, retaining coefficients for 11 genes. The optimal parameter λ=0.040 was determined through coefficient distribution analysis (**Figure 8A**). A lollipop plot displayed the coefficients of the 11 genes obtained (**Figure 8B**). Using the median of each dataset, we divided the training and validation sets into high-risk and low-risk groups. Throughout both the training set TARGET-OS and the validation sets GSE21257 and GSE16091, the low-risk cohort consistently demonstrated markedly superior survival prognosis compared to the high-risk cohort, with the disparity in survival rates escalating over time. ROC curve analysis revealed that the area under the curve (AUC) for all three datasets at 1, 3, and 5 years was greater than 0.8, indicating good diagnostic performance of the model at these time points (**Figure 8C**).

### 3.5 Further analysis of the model and drug prediction

**Figure 9:**
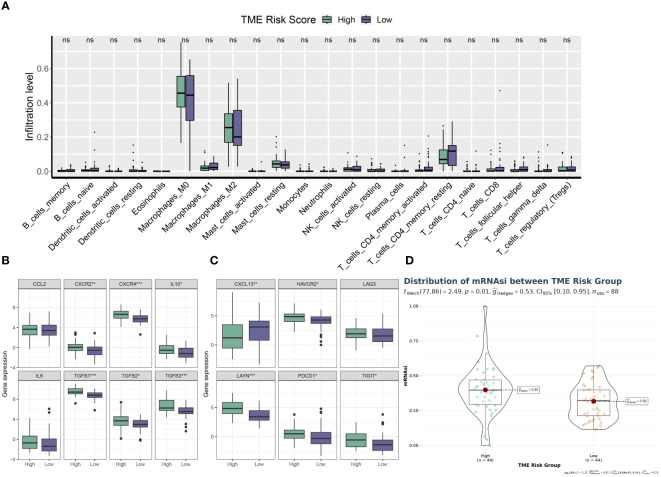
TME phenotypes between risk groups. **(A)** Box plot illustrating the distributions of 22 immune cell subsets determined by CIBERSORT between two risk groups. **(B, C)** Box plot illustrating the expression profiles of M2 polarization regulators **(B)** and TEXterm features **(C)** between two risk groups. **(D)** Violin plot displaying the mRNAsi index between two risk groups. * p<0.05; ** p<0.01; *** p<0.001; **** p<0.0001.

**Figure 10:**
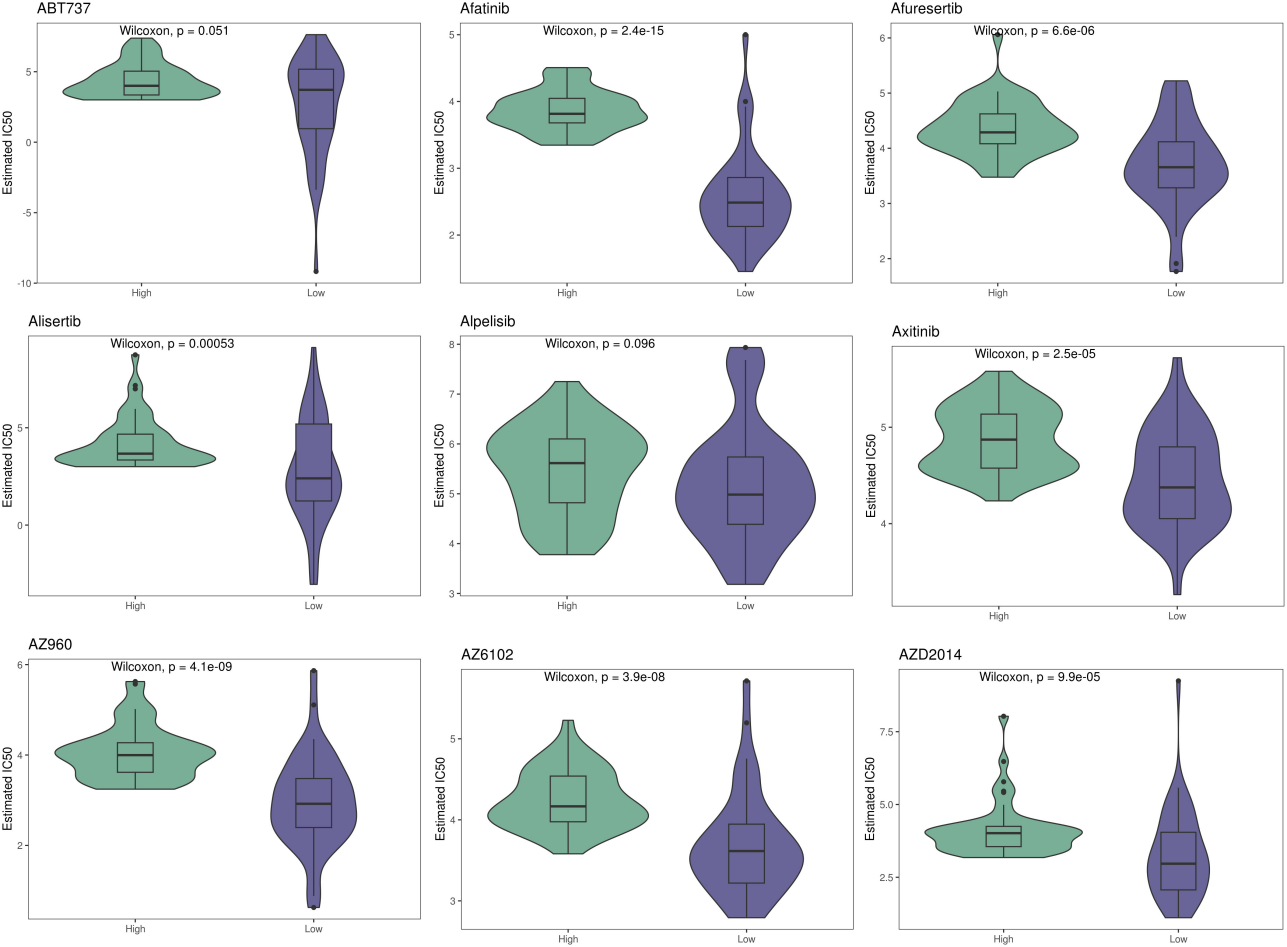
Therapeutic sensitivity between two risk groups.

**Figure 11:**
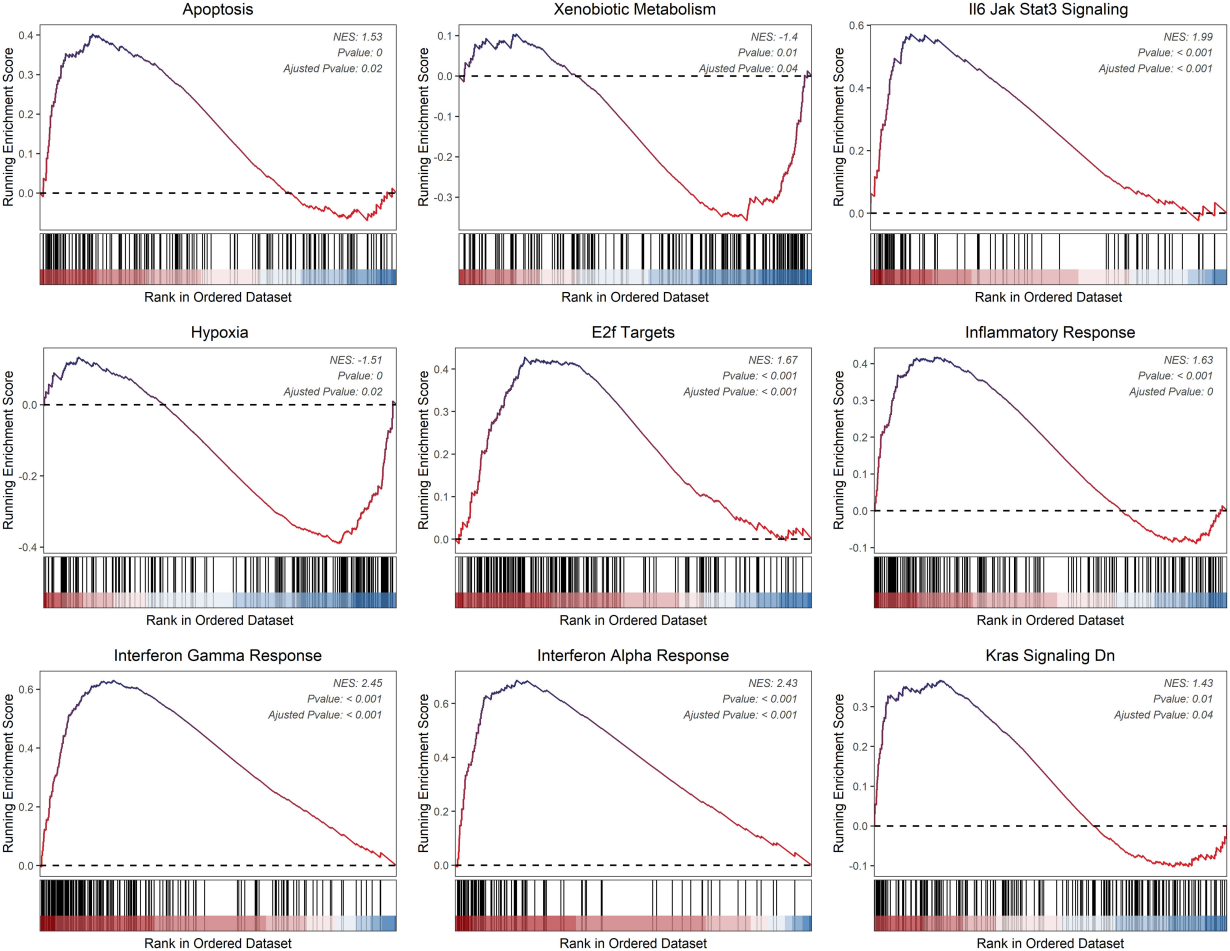
Dysregulated cancer hallmarks between two risk groups.

Using CIBERSORT, we conducted immune cell infiltration analysis to screen for relevant immune cell subgroups. Interestingly, we did not observe any significant differences between the two groups in terms of various immune cell populations (**Figure 9A**). We generated box plots to show the expression differences of M2 markers and exhausted T cell markers between the high and low-risk groups. In the high-risk group, most markers had higher expression levels compared to the low-risk group, except for CXCL13, which was lower in the high-risk group. (**Figures 9B, C**). Violin plots visually depicted the disparity in cellular stemness analysis between the high and low-risk groups based on the mRNAsi index, revealing a higher mRNAsi index in the high-risk group (p=0.01, **Figure 9D**). We also conducted drug sensitivity analyses for nine selected drugs, comparing the high and low-risk groups. Violin plots demonstrated higher IC50 values in the high-risk group, indicating reduced drug sensitivity (**Figure 10**). Differential gene expression analysis between the two risk groups was followed by Gene Set Enrichment Analysis (GSEA) to identify commonly dysregulated pathways. The high-risk group exhibited upregulation in most frequently altered tumor pathways (**Figure 11**).

### 3.6 Single-cell sequencing analysis

**Figure 12:**
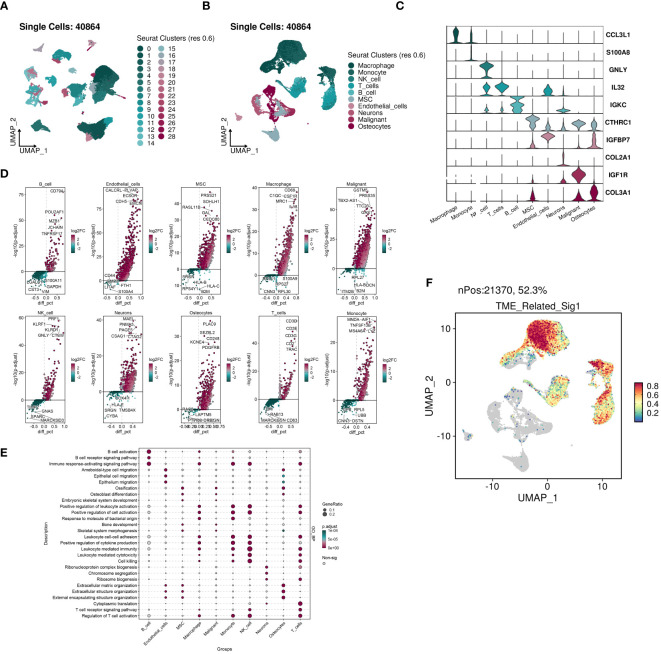
The highly activated TME-related signature in scRNA-seq datasets of OS. **(A)** UMAP visualization of 40864 cells from four public OS scRNA-seq cohorts. **(B)** 10 major cell types were manually annotated. **(C)** Vlnplots illustrating the expression values of cell type-specific markers. **(D)** Volcano plots illustrating the top five labeled markers upregulated (colored in red) or downregulated (colored in blue) in each cell cluster. **(E)** Dot plot showing the enriched GO_BP terms of each cell cluster. **(F)** The signature genes expression at single cell level determined by AddModuleScore() function in Seurat.

We conducted an analysis of the acquired single cell sequencing data. We used UMAP dimensionality reduction clustering on the integrated single-cell data (**Figure 12A**), and ten cell subgroups annotated and visualized ten cell subgroups based on cell-specific markers (**Figure 12B**). We examined the expression of various genes across the ten cell subgroups, revealing higher expression of COL3A1 in MSCs, Malignant cells, and Osteocytes, with significant upregulation of IGF1R in Malignant cells (**Figure 12C**).Utilizing SingleR for automated annotation combined with copycat for malignant cell identification, we computed the upregulated and downregulated genes in each cell subgroup and presented volcano plots showing the top five upregulated and downregulated genes (**Figure 12D**). GO_BP analysis indicated significant upregulation of multiple pathways in Macrophages, monocytes, T cells, NK cells, and B cells (**Figure 12E**). Furthermore, we evaluated the distribution of prognostic model scores within the single-cell dataset, revealing a significant regional pattern (**Figure 12F**).

### 3.7 Knockdown of ACSL5 inhibited the proliferation, invasion, and migration of OS cells

**Figure 13:**
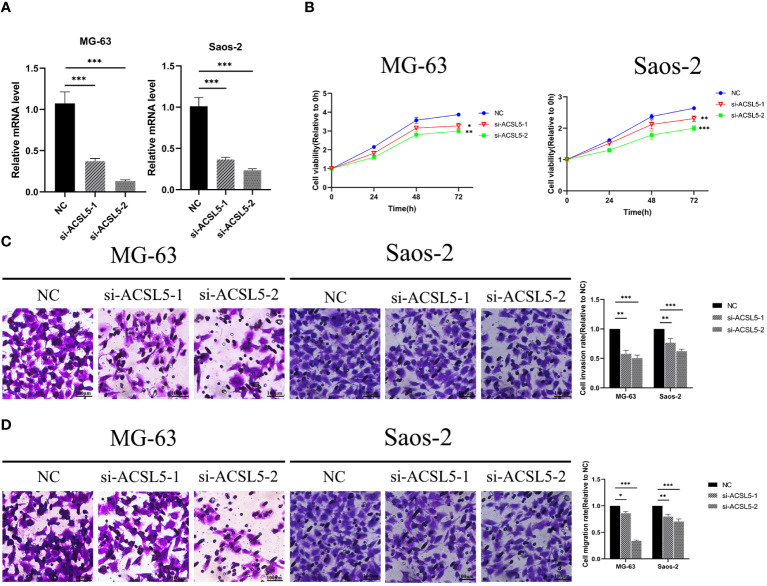
**(A)** The downregulation of ACSL5 expression in MG63 and Saos-2 cells was confirmed by RT-qPCR using two distinct siRNA sequences. **(B)** Differential knockdown efficacy of ACSL5 expression in MG-63 and Saos-2 cells was observed between the two siRNA sequences. **(C)** Following knockdown of ACSL5 using both sets of sequences, differential invasion capacities were observed in MG-63 and Saos-2 cells. **(D)** Subsequent to the knockdown of ACSL5 expression using both sets of sequences, differential migration capacities were observed in MG-63 and Saos-2 cells. * p<0.05; ** p<0.01; ****p<0.0001.

**Figure 14:**
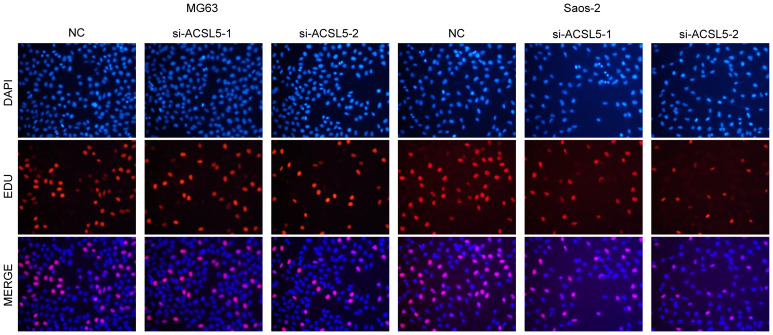
EdU assay between the control group and ACSL5 knockdown cells.

**Figure 15:**
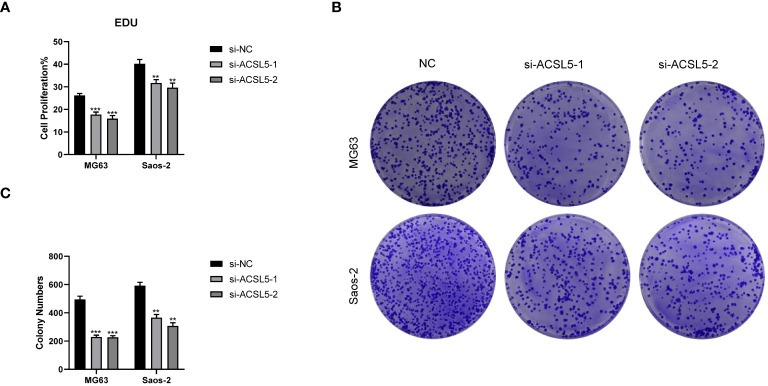
Colony formation experiments between the control group and ACSL5 knockdown cells. **(A)** The statistical data of the EDU assay. **(B, C)** The colon formation assay of ACSL5. ** p<0.01; *** p<0.001.

To assess the potential impact of ACSL5 inhibition on the aggressive behavior of OS cells, we used siRNA to downregulate ACSL5 expression in MG63 and Saos-2 cells. We selected two siRNA sequences and confirmed by RT-qPCR. As depicted in **Figure 13A**, both sets of sequences effectively reduced ACSL5 expression in MG-63 and Saos-2 cells, with si-ACSL5–2 demonstrating notably superior knockdown efficacy compared to si-ACSL5–1. As illustrated in **Figure 13B**, ACSL5 knockdown significantly curtailed cell proliferation within 72 hours. **Figures 13C, D** further demonstrated that ACSL5 knockdown markedly impeded the invasion and migration of both MG-63 and Saos-2 cells. We further used more experiments to explore the effect of ACSL5 on proliferation. EdU staining results indicated that the proliferation capacity of both MG63 and Saos-2 knockdown groups was significantly lower compared to the NC group, indicating inhibited cell proliferation post-knockdown (**Figures 14**, **15A**). Colony formation assay results revealed that the proliferation capacity of the NC group was significantly superior to that of the knockdown groups (**Figures 15B, C**). Hence, our findings suggest that silencing ACSL5, a pro-oncogene, could attenuate the oncogenic behaviors of proliferation, migration, and invasion in OS cells.

## 4 Discussion

OS stands as the prevailing primary malignant bone tumor, presenting with the highest occurrence in children and adolescents, thereby securing the third position among malignant tumors within this age group. Though rare, OS has a poor prognosis. Surgery is the main curative treatment, but patients undergoing only surgery have a survival rate of about 15%. The 5-year survival rate is over 78% for localized OS but drops to 25% for metastatic or recurrent cases. For those unable to have surgery, radiotherapy is effective for local control and symptom relief. However, advanced-stage OS is highly invasive and has a poor prognosis. Therefore, investigating the mechanisms underlying OS-related genes, particularly those implicated in its elevated metastatic potential and recurrence rates, deciphering pivotal biological markers, and exploring essential target genes emerge as critical endeavors for enhancing the diagnosis, treatment, and prognosis of OS.

We conducted a multi-layered analysis using various types of transcriptome data downloaded from multiple public databases. We analyzed the expression differences of four TME-related gene sets between tumor and normal groups in the integrated bulk matrix obtained from the TARGET-OS and GTEx datasets. We performed unsupervised clustering analysis on tumor tissues, selecting k=2 as the optimal number based on matrix plots, CDF curves, and PAC scores. We calculated GSVA scores for four TME-related gene sets in the TARGET-OS dataset and conducted univariate Cox analysis. C2 had higher scores than C1 in most TME scoring items. The analysis revealed TMEScoreA_CIR and TMEscoreA_plus as prognostic risk factors. We explored the differences between the two clusters through Kaplan-Meier curves, distribution of clinical pathological information, analysis of immune cell infiltration, prediction of tumor immune escape, and abundance of immune inhibitory cells. The results showed that C1 had a poorer prognosis but was more relevant to OS treatment. We analyzed drug sensitivity in C1 and C2 using four drugs, finding that C2 was more sensitive to them, though this needs further validation. We identified DEGs between the clusters and performed GO enrichment analysis and GSEA.

Performing WGCNA on the TARGET-OS dataset, we obtained the optimal soft threshold power=3 to construct a co-expression network and partition gene modules. We identified the key module, MEbrown, and filtered critical genes with MM > 0.6 and GS > 0.3, then performed GO enrichment analysis. We used TARGET-OS as the training set and two GEO datasets as validation sets, defining two risk groups based on the median score of each dataset. Using LASSO and multiple regression analysis, we built a prognostic model and identified 11 genes: ALOX5AP, CD37, BIN2, C3AR1, HCLS1, ACSL5, CD209, FCGR2A, CORO1A, CD74, and CD163. Among these, ALOX5AP is a crucial enzyme that converts arachidonic acid to leukotrienes, serving as an important immunomodulatory lipid mediator. Diseases associated with ALOX5AP include stroke, ischemia, and myocardial infarction (26). Prior research also suggests widespread expression of ALOX5AP in 20 different types of epithelial cancer cell lines, implicating its potentially crucial role in influencing cancer patient prognosis (27). CD37, encoding a protein member of the transmembrane 4 superfamily, also known as the tetraspanin family, is associated with osteogenesis imperfecta, III-type, and mantle cell lymphoma, playing a critical role in regulating tumor onset and progression (28). CD37 serves as a significant immune marker in various immune cells (e.g., T cells, B cells, and macrophages), with high expression possibly indicating adequate filtration and immune competence in the tumor microenvironment (29). BIN2, encoding a cytoplasmic protein, influences podosome formation, movement, and phagocytosis through interactions with the cell membrane and cytoskeleton (30). Meanwhile the role of BIN2 in cancer remains yet unclear, TCGA studies have observed an association between upregulated BIN2 and favorable survival outcomes in all cervical, endometrial, breast, and ovarian cancers (31). C3AR1, as the orphan G protein-coupled receptor for the allergic toxin C3a released during complement system activation, plays a crucial role in immune responses, particularly implicated in immune infiltration in sepsis (32). Endothelial C3AR1 regulates vascular inflammation in aging or neurodegenerative diseases (33). HCLS1, containing a Src homology 3 (SH3) domain, facilitates the activation of receptor tyrosine kinases (34). Levels of HCLS1 were linked to chronic lymphocytic leukemia, though its role in cancers, particularly OS, remains unclear. ACSL5, a mitochondrial enzyme, aids in the synthesis of long-chain fatty acyl-CoA and induces cellular apoptosis. Its predominant isoform in mitochondrial cardiolipin biosynthesis might also support cancer cell survival (35, 36). Previous studies suggest its crucial role in the malignant progression and metastasis of gliomas (37). CD209, also known as DC-SIGN, belongs to the C-type lectin superfamily primarily expressed in dendritic cells (38). CD209 binds Lewis antigens highly expressed in cancers, facilitating T-cell priming and initiating immune cascades (39). FCGR2A, a member of the immunoglobulin Fc receptor gene family found on various immune response cells, participates in immune surveillance and validation (40). Its association with the pharmacodynamics of monoclonal antibodies varies across different cancer types like colorectal, breast, and metastatic squamous cell carcinoma of the head and neck (41). CORO1A, encoding a member of the WD repeat protein family, is involved in multiple cellular processes, including cell cycle, apoptosis, signal transduction, and gene regulation (42). Previous studies identified CORO1A as a pro-proliferative target in breast cancer cells (42). CD74, also known as invariant chain, acts as an MHCII chaperone crucial in antigen presentation (43). Research suggests its diverse roles within cells and the entire immune system, highlighting its potential as a therapeutic target for cancer and autoimmune diseases (44). CD163, an abundant endocytic receptor for various ligands, is particularly enriched in the inflammatory and tumor microenvironments with CD163^+^ macrophages (45). Studies indicate CD163-positive M2-polarized macrophages as robust biomarkers for diagnosis and stratification of OS patients (46). We divided the cohorts into high and low-risk groups based on the median scores of each dataset. Survival analysis showed a poorer prognosis for the high-risk group, while ROC curve analysis confirmed the model’s strong performance at 1, 3, and 5 years.

Immune cell infiltration analysis identified relevant immune cell subtypes. Box plots illustrated differential expression of M2 and exhausted T cell markers between the two risk groups, with significantly higher expression observed in most of the high-risk group. Both markers are associated with tumor immune suppression, indicating a poorer immune microenvironment in the high-risk group (47). Violin plots depicted higher mRNAsi indices in the high-risk group. Sensitivity analysis to nine drugs indicated lower drug sensitivity in the high-risk group. Differential gene expression analysis between the two risk groups, followed by GSEA analysis of dysregulated pathways, revealed upregulation of numerous common tumor pathways in the high-risk group, suggesting activation of multiple tumor progression pathways and resistance to drug therapy and immune cell cytotoxicity. The malignant characteristics of tumors in the high-risk group are multifaceted and interrelated. Further exploration of tumor characteristics is necessary to develop targeted therapies for such patients. UMAP dimensionality reduction clustering of integrated single-cell data annotated ten cell subtypes based on cell-specific markers, with subsequent analysis of gene expression within each subtype. We annotated and identified malignant cells and calculated upregulated and downregulated genes in each subtype. GO_BP analysis showed significant pathway upregulation in certain cell subtypes. Using Seurat, we evaluated our prognostic model’s activity in single-cell datasets, confirming its effectiveness.

ACSL5 belongs to an activating enzyme family of long-chain fatty acids (LCFAs), the role of which are not well understood. ACSL5 expression correlates with improved survival in lung cancer patients, and plasma EA levels predict immunotherapy success. Targeting ACSL5 may enhance immunotherapy by reprogramming antigen presentation (48). Research indicates that the protein PPARGC1A is linked to the development of hepatocellular carcinoma (HCC), although its exact functions and related pathways are not fully understood. PPARGC1A is under-expressed in HCC and correlates with a poorer prognosis. As regard to the underlying mechanisms, a PPARGC1A/BAMBI/ACSL5 axis is found to be responsive to hypoxia (49). In an effort to identify crucial biomarkers for pancreatic cancer prognosis, a study discovered a total of four genes, ACSL5, SLC44A4, LOX, and TOX3, showing correlation with PFS as indicated by qPCR and immunohistochemical staining. Further analysis revealed that differentiation status, tumor stage, LOX expression, and ACSL5 expression were independent factors predicting prognosis (50). In the context of OS, as one of ferroptosis-related genes, ACSL5 was integrated into a prognostic model for OS patient prognosis (51). However, the mechanic role of ACSL5 in the OS carcinogenesis remains to be further clarified. In our study, we validated that silencing ACSL5 (a pro-oncogene) via cell culture, siRNA transfection, RT-qPCR, cell proliferation assays, and cell migration and invasion assays reduced oncogenic behaviors like proliferation, migration, and invasion in OS cells.

## 5 Conclusion

This study conducted an in-depth analysis of the TME in OS, revealing two significantly distinct subgroups. Our prognostic model, based on eleven key genes (ALOX5AP, CD37, BIN2, C3AR1, HCLS1, ACSL5, CD209, FCGR2A, CORO1A, CD74, CD163), demonstrated good performance in predicting patient survival and disease progression. Additionally, we conducted immune analysis, drug sensitivity analysis, and gene enrichment analysis, providing new insights and theoretical foundations for the treatment and drug development of OS patients. Single cell sequencing analysis further revealed the expression profiles of cell subgroups, deepening our understanding of the immune microenvironment in OS. In summary, our study provides valuable insights and guidance for improving the prognosis of OS patients. It highlights key areas for optimizing treatment strategies and supports the development of more effective drugs. By identifying crucial genes and pathways, our research lays the groundwork for targeted therapies and personalized medicine approaches in OS, ultimately aiming to enhance patient outcomes and survival rates.

## Data availability statement

The datasets presented in this study can be found in online repositories. The names of the repository/repositories and accession number(s) can be found in the article/supplementary material.

## Ethics statement

Ethical approval was not required for the studies on humans in accordance with the local legislation and institutional requirements because only commercially available established cell lines were used. Ethical approval was not required for the studies on animals in accordance with the local legislation and institutional requirements because only commercially available established cell lines were used.

## Author contributions

SS: Conceptualization, Data curation, Writing – original draft. LZ: Conceptualization, Formal analysis, Investigation, Project administration, Writing – review & editing. XG: Conceptualization, Data curation, Writing – original draft.
